# Analysis of the Generation of Harmful Aldehydes in Edible Oils During Sunlight Exposure and Deep-Frying Using High-Field Proton Nuclear Magnetic Resonance Spectroscopy

**DOI:** 10.3390/foods14030513

**Published:** 2025-02-05

**Authors:** Anna Meike Freis, Sahithya Phani Babu Vemulapalli

**Affiliations:** Institute for Chemistry and Biology of the Marine Environment (ICBM), School of Mathematics and Science, Carl von Ossietzky Universität Oldenburg, Ammerländer Heerstraße 114–118, 26129 Oldenburg, Germany; anna.meike.freis@uni-oldenburg.de

**Keywords:** nuclear magnetic resonance (NMR), high-field ^1^H NMR spectroscopy, edible oils, fatty acids (FAs), photodegradation, thermal degradation, lipid oxidation products (LOPs), hydroperoxides, toxic aldehydes

## Abstract

Edible oils are essential dietary components that provide crucial micronutrients. However, their quality can deteriorate during frying—a common cooking method—and with prolonged light exposure due to chemical reactions such as hydrolysis, oxidation, and polymerization. These processes lead to the formation of harmful compounds, particularly aldehydes. This study investigates how thermal and light exposure impact the chemical composition of five widely used edible oils: olive, rapeseed, sunflower, sesame, and peanut oils. For the thermal treatment, the oils were heated to 190 ± 5 °C in a commercial fryer, with samples taken at the start and after 10 min and 60 min of heating, while intermittently frying chicken nuggets to simulate typical frying conditions. For the light exposure treatment, the oil samples were exposed to direct sunlight for 3 and 8 h, with control samples being collected beforehand. The oil composition was analyzed using an advanced 800 MHz nuclear magnetic resonance (NMR) instrument with a triple-resonance inverse cryoprobe, providing high sensitivity and resolution. The results revealed a significant increase in various aldehyde compounds in all oils under both thermal and light exposure conditions. Notably, this study identified the generation of genotoxic and cytotoxic *α*,*β*-unsaturated aldehydes, including 4-hydroperoxy-(*E*)-2-alkenals, 4-hydroxy-(*E*)-2-alkenals, and 4,5-epoxy-(*E*)-2-alkenals. Given the established association of aldehydes with health risks, including cancer, Alzheimer’s, and Parkinson’s diseases, these findings highlight the importance of monitoring oil degradation during cooking and the appropriate storage of oils to minimize light exposure to reduce potential health risks.

## 1. Introduction

Edible oils play a crucial role in the human diet, providing essential fatty acids and micronutrients that support overall health [1]. They are widely used in cooking methods such as deep-frying, grilling, baking, and sautéing [2]. Among these, deep-frying is particularly common in both domestic kitchens and commercial food establishments, where oils are heated to temperatures as high as 180 °C [3,4]. However, when oils are exposed to high heat, typically exceeding their smoke points, they undergo a series of chemical reactions including hydrolysis, oxidation, isomerization, polymerization, and degradation [5,6]. These reactions significantly alter the oils’ color, flavor, nutritional properties, and molecular composition.

To reduce costs, cooking oils are often reused in deep-frying for extended periods [7], which severely degrades their quality. This repeated use leads to the formation of potentially harmful compounds such as polycyclic aromatic hydrocarbons (PAHs) [8,9] and aldehydes [10,11,12]. The thermal decomposition of cooking oils initially produces primary lipid oxidation products (LOPs) from unsaturated fatty acids (UFAs), which subsequently degrade into highly toxic aldehydes [13,14,15,16]. Aldehydes, particularly *α*,*β*-unsaturated aldehydes, are exceptionally reactive, readily interacting with essential biomolecules like DNA and causing significant damage. Their mutagenic, genotoxic, and carcinogenic effects are well documented [17], with additional health risks including cancer, cardiovascular diseases, and neurological disorders [15,18].

In addition to deep-frying, the improper handling and storage of edible oils exacerbate their degradation through oxidation and related chemical changes [19]. Factors such as light exposure, temperature, oxygen concentration, water content, and metal contamination affect the oil’s stability and quality. In particular, sunlight accelerates decomposition via photooxidation. During this process, ultraviolet (UV) and visible light photons interact with unsaturated fatty acids, generating free radicals and reactive oxygen species (ROS). These reactions lead to oxidative degradation, resulting in the formation of primary oxidation products, such as peroxides and secondary oxidation products, including aldehydes and ketones. These degradation products reduce the nutritional values, shelf life, and sensory qualities of oils [14,20].

The susceptibility of edible oils to photooxidation depends on their degree of unsaturation and environmental storage conditions. Oils that are rich in polyunsaturated fatty acids (PUFAs) are particularly vulnerable due to their high reactivity with oxygen and light [21]. Naturally occurring or intentionally added antioxidants, such as tocopherols, tocotrienols, carotenoids, phenolic compounds, and sterols, play a vital role in preventing or slowing down the oxidation of edible oils, thereby enhancing their durability and shelf life [14,22]. In the presence of light, pigments like chlorophyll act as photosensitizers by generating singlet oxygen (¹O_2_), which accelerates the oxidation process in edible oils [14]. Conversely, in the absence of light, chlorophyll compounds help reduce the rate of aut-oxidation in oils [14]. Photooxidation is a free radical chain reaction involving initiation, propagation, and termination steps, where lipid alkyl radicals are generated by the removal of hydrogen atoms from fatty acids or acylglycerols. Factors such as heat, metal catalysts, and light further accelerate this process. Hydrogen atoms adjacent to double bonds, especially those between two double bonds, are most susceptible to oxidation [14]. Understanding the mechanisms and effects of photooxidation is critical for optimizing storage conditions and developing protective strategies to preserve oil quality.

Repeated heating and improper storage of edible oils pose significant health risks, yet these practices remain widespread. Therefore, understanding how cooking conditions, light exposure, and storage practices affect oil degradation is crucial for mitigating these risks. Advanced analytical techniques are essential for characterizing the molecular changes in oils under these conditions. In this regard, nuclear magnetic resonance (NMR) spectroscopy [23,24], gas chromatography–mass spectrometry (GC-MS) [25], Fourier-transform infrared (FT-IR) spectroscopy [26], UV–Vis spectroscopy [27,28], and Raman spectroscopy [29,30] are frequently employed for analyzing the degradation of oils. Among these, proton (^1^H) NMR spectroscopy is particularly effective. This non-destructive and highly precise method allows for the detection of secondary lipid oxidation products, such as aldehydes and ketones, while simultaneously tracking changes in fatty acid profiles [23,24].

Numerous studies highlight the effectiveness of ^1^H NMR in quantifying the accumulation of toxic aldehydes in heated oils [10,11,31,32], showing a linear increase in both saturated and unsaturated aldehydes over time. However, one-dimensional (1D) ^1^H NMR spectroscopy at low magnetic fields has limitations, including poor resolution due to narrow chemical shift dispersion (approximately 10 ppm) and low sensitivity for detecting secondary lipid oxidation products, such as aldehydes.

To address these challenges, this study investigates the effects of thermal and photodegradation on five commonly used edible oils—olive, rapeseed, sunflower, sesame, and peanut—using a high-field 800 MHz NMR instrument equipped with a helium-cooled triple-resonance inverse cryoprobe. This advanced setup offers enhanced resolution and sensitivity compared to conventional analytical technologies, enabling the detection of subtle compositional changes in oils. By analyzing the degradation processes under repeated heating and light exposure, this study aims to elucidate the molecular transformations in these oils. The findings emphasize the need for improved guidelines on the safe use and storage of edible oils to preserve their quality and minimize health risks.

## 2. Materials and Methods

Five edible oils—olive, rapeseed, sunflower, sesame, and peanut—were purchased from a local supermarket in Germany and used as received. These oils were selected based on their distinct acyl group composition to investigate the evolution of their molecular composition under thermal and photo stresses. The oils were labeled by the manufacturer as follows: extra virgin olive oil, rapeseed oil with omega-3 fatty acids, cold-pressed sunflower oil (native, rich in vitamin E), cold-pressed sesame oil (rich in unsaturated fatty acids), and peanut oil. To prevent light exposure, all oils were purchased in amber-colored bottles and stored in a dark place before and after the experiments. Deuterated chloroform (CDCl_3_) and 5 mm NMR tubes were obtained from Deutero GmbH, Kastellaun, Germany. A Silvercrest^®^ Kitchen Tools mini deep fryer was purchased from a local supermarket in Germany. A MixcMax kitchen thermometer (Dongguan Challian Electronic Technology, Dongguan, China) (temperature range: −50 to +300 °C) was used for temperature monitoring. Chicken nuggets were also obtained from a local supermarket in Germany.

### 2.1. Sunlight Treatment

All five oils—olive, rapeseed, sunflower, sesame, and peanut—were subjected to sunlight treatment. For the photodegradation experiments, 60 µL of oil, directly from the bottle, was dissolved in 540 µL of CDCl_3_ to achieve a final volume of 600 µL, and the mixture was transferred to 5 mm NMR tubes. These NMR tubes were then used for the sunlight treatment of the oils. The initial ^1^H NMR spectra of all five oils were recorded on freshly prepared samples as reference spectra (t0). The NMR tubes were subsequently placed in bright sunlight at 26 ± 2 °C under moderate-to-high UV index (values in the range of 5–6) conditions. ^1^H NMR spectra were recorded after 3 h (t1) and 8 h (t2) of sunlight exposure.

### 2.2. Deep-Frying

All oils—rapeseed, sunflower, sesame, and peanut—were subjected to deep-frying experiments, except for the olive oil. Extra virgin olive oil was not included in the deep-frying experiments, as it is not commonly used for deep-frying due to its high cost and low smoke point. Extra virgin olive oil is highly prone to degradation at higher temperatures, leading to the formation of toxic fumes and harmful substances. In a study by Guillén and Uriarte [33], the evolution of extra virgin olive oil subjected to deep-frying at 190 °C was investigated using ^1^H NMR spectroscopy. The study revealed the formation of toxic aldehydes, such as alkanals, (*E*)-2-alkenals, (*E,E*)-2,4-alkadienals, and 4-oxoalkanals, among others. Approximately 1 L of oil was added to the mini deep fryer. Before heating, a 1 mL oil aliquot was taken as the control sample (t0). The fryer temperature was set to 190 °C and monitored using the MixcMax kitchen thermometer. Chicken nuggets were fried intermittently during a 60 min continuous deep-frying session to mimic commercial fast-food frying practices. Oil samples were taken at 10 min (t1) and 60 min (t2). Aliquots of 1 mL of oil were transferred into amber-colored vials to prevent light exposure. The oil samples were allowed to cool to room temperature and filtered through 0.2 µm Whatman filters. The filtered oil samples were used to prepare the NMR samples.

### 2.3. NMR Sample Preparation

For the deep-frying and sunlight treatment experiments, 60 µL of filtered oil was dissolved in 540 µL of CDCl_3_ to achieve a final volume of 600 µL, which was then transferred to 5 mm NMR tubes.

### 2.4. NMR Spectroscopy

NMR spectra were acquired using a Bruker Avance Neo 800 MHz instrument (Ettlingen, Germany) equipped with a helium-cooled 5 mm TCI cryoprobe. Samples were allowed to reach thermal equilibrium before acquisition. Locking, tuning and matching, and shimming were performed for each sample. The one-dimensional ^1^H NMR spectra were recorded using the Bruker standard *zg* pulse sequence. A total of 64 k points were acquired over a 16 kHz spectral width, with an acquisition time of 2 s and a relaxation delay of 3 s, resulting in a 5 s repetition time. Each spectrum was acquired with 64 scans, requiring 6 min per sample. The ^1^H chemical shift range covered was from 16 to −4 ppm, with the transmitter frequency offset set at 6 ppm. Spectra were processed using an exponential multiplication window function with a 0.3 Hz line-broadening. The residual CDCl_3_ signal at 7.26 ppm was used as the chemical shift reference. The data acquisition and processing were performed using Topspin 4.4.1 software (Bruker, Ettlingen, Germany).

## 3. Results and Discussion

### 3.1. The Molecular Composition of Edible Oils Before Treatment

**Figure 1:**
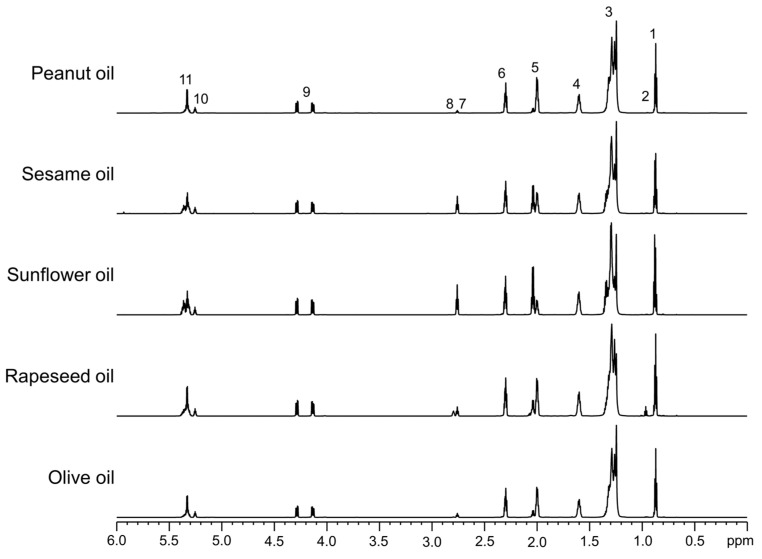
^1^H NMR spectra of the olive, rapeseed, sunflower, sesame, and peanut oils were recorded in CDCl_3_ using an 800 MHz instrument. The functional group assignments are provided in Table 1.

The ^1^H NMR spectra of the olive (O), rapeseed (R), sunflower (SF), sesame (S), and peanut (P) oils display prominent signals corresponding to the protons of triglycerides, the primary lipid components of these oils (Figure 1). The key structural groups of the oils were assigned based on their proton chemical shifts and *J*-coupling multiplicity patterns, as previously reported [31] (Table 1).

While the ^1^H NMR spectra of all five oils appear broadly similar, a closer examination of specific signals provides insights into the relative abundance of linolenic, omega-3, linoleic, oleic, monounsaturated, and saturated acyl groups. The following equations [32] are used to derive the molar percentages of various acyl groups in the edible oils:

$Linolenic\;groups,\;Ln\;\left(\%\right)=100\left(A_{8}/3A_{9}\right)$

$Linoleic\;groups,\;L\;\left(\%\right)=100\left(2A_{7}/3A_{9}\right)$

$Oleic\;\left(or\;monounsaturated\right)\;groups,\;O\;\left(or\;MU\right)\left(\%\right)=100\left[\left(A_{5}-2A_{7}-A_{8}\right)/3A_{9}\right]$

$Saturated\;groups,\;S\;\left(\%\right)=100\left[1-\left(A_{5}/3A_{9}\right)\right]$

where A5, A7, A8, and A9 are the areas of signal 5 (mono-allylic protons of all unsaturated acyl groups), signal 7 (bis-allylic protons of linoleic groups), signal 8 (bis-allylic protons of linolenic groups), and signal 9 due to the methylene protons of the glycerol backbone of triglycerides, respectively. These signals are indicated in Table 1 and Figure 1. The calculated molar percentages of the acyl groups align with the expected values for edible oils of this type.

A distinctive feature of rapeseed oil, compared to the other oils, is the prominent bis-allylic =CH-CH_2_-CH= signal at 2.79 ppm and the terminal methyl signal at 0.96 ppm, indicating a high abundance of linolenic or ω-3 acyl groups. This finding corroborates the information provided on the rapeseed oil label, which describes it as "rapeseed oil with omega-3 fatty acids”. The abundance of linolenic acid is highest in rapeseed oil, followed by olive, sesame, sunflower, and peanut oils (R > O > S > SF > P) (Table 2). The intensity of the terminal methyl signal for ω-3 fatty acids further supports this trend. Additionally, sunflower oil is particularly rich in linoleic acyl groups, with the oils ranked in the following order: SF > S > R > O > P (Table 2). The abundance of oleic or monounsaturated acyl groups follows the order: P > O > R > S > SF (Table 2). Olive and sesame oils contain relatively high levels of saturated acyl groups, followed by peanut and sunflower oils, while rapeseed oil has the lowest levels of saturated acyl groups.

### 3.2. Generation of Primary Lipid Oxidation Products (LOPs) in Oils During Sunlight Irradiation

**Figure 2:**
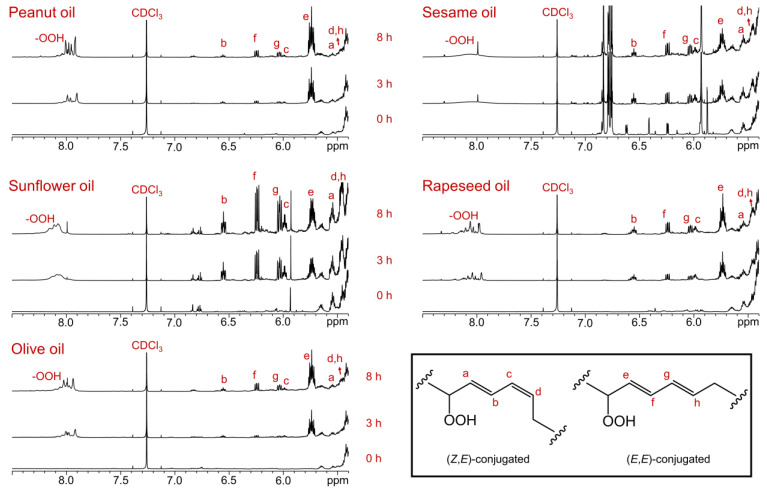
Olefinic and hydroperoxide proton regions of the ^1^H NMR spectra of oils recorded in CDCl_3_ using an 800 MHz instrument. Each panel is labeled with the corresponding oil type. The bottom, middle, and top spectra represent oil samples collected after 0, 3, and 8 h of sunlight exposure, respectively, at 26 ± 2 °C under moderate to high UV index (in the range 5–6) conditions. Compared to untreated control samples, the sunlight-exposed oil samples exhibit the formation of (*Z*,*E*)- and (*E*,*E*)-conjugated double bonds associated with hydroperoxides [31,35]. The chemical structures of primary lipid oxidation products, such as hydroperoxides, are shown with atom labeling that corresponds to the proton NMR signal assignments.

All five oils—olive, rapeseed, sunflower, sesame, and peanut—were exposed to sunlight for 3 and 8 h at 26 ± 2 °C under moderate-to-high UV index (in the range 5–6) conditions. The formation of new compounds in these oils was monitored by recording their ^1^H NMR spectra at specific time intervals. The ^1^H NMR spectra of the oils at the initial time point (0 h of sunlight exposure) were used as references to track molecular changes during photooxidation. At 0 h, no characteristic signals were observed in the chemical shift region of 8.5–5.4 ppm (Figure 2, 0 h).

After 3 h of sunlight exposure, new signals appeared in the regions of 6.6–5.4 ppm and 8.5–7.8 ppm, corresponding to olefinic and hydroperoxide protons, respectively (Figure 2, 3 h). The intensity of these signals increased further after 8 h of sunlight exposure (Figure 2, 8 h). In the spectral range of 8.5–7.8 ppm, a residual signal from CDCl_3_ at 7.26 ppm, used as an internal chemical shift reference, was also observed. The broad signals in this region, attributed to hydroperoxide protons, are labeled as -OOH. The characteristic olefinic proton signals, labeled as a–h, were consistent with the assignments shown on the chemical structures (Figure 2). The initial step of photooxidation in the fatty acid (FA) acyl groups of the oils resulted in the formation of primary lipid oxidation products, namely hydroperoxides [15,34]. This was evidenced by the broad signals in the 8.5–7.8 ppm region. Based on the chemical shifts and J-coupling patterns of the olefinic protons, the primary oxidation products were identified as *cis*,*trans* (*Z*,*E*)- and *trans*,*trans* (*E*,*E*)-conjugated dienes containing hydroperoxides (Figure 2 and Table 3). The proton signal at 6.55 ppm (ddd, J = 15.2, 11.0, and 4.0 Hz), labeled as “b”, indicated the formation of hydroperoxides with (*Z*,*E*)-conjugated diene structures. The integral of this olefinic proton signal revealed the relative formation of (*Z*,*E*)-conjugated diene hydroperoxides in the following order: SF > S > R > O > P. Similarly, the integral of the proton signal at 6.24 ppm (dd, J = 15.2 and 10.4 Hz), labeled as “f”, corresponding to (*E*,*E*)-conjugated diene hydroperoxides, showed the same relative abundance: SF > S > R > O > P.

Overall, the sunflower oil demonstrated the highest proportion of hydroperoxides after 8 h of sunlight exposure, which is consistent with its high content of linoleic acyl groups (Table 2), known to be highly prone to photooxidation. The sesame and rapeseed oils followed, exhibiting increased production of conjugated diene hydroperoxides. This can be attributed to the abundance of linoleic acid in sesame oil and both linolenic and linoleic acids in rapeseed oil (Table 2). In contrast, the olive and peanut oils produced relatively lower amounts of primary oxidation products, likely due to their lower content of linolenic and linoleic acyl groups (Table 2) in their lipid composition. Additionally, the photooxidation of edible oils may also be influenced by the presence of photosensitizers, such as chlorophyll, and by naturally occurring or externally added antioxidants.

### 3.3. Sunlight-Mediated Formation of Aldehyde Compounds in Oils

**Figure 3:**
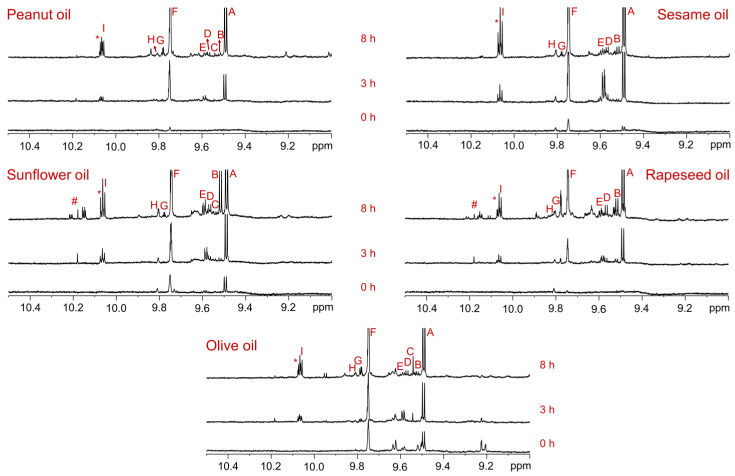
Aldehyde proton region of ^1^H NMR spectra of oils recorded in CDCl_3_ using an 800 MHz instrument. Each panel is labeled with the corresponding oil type. The bottom, middle, and top spectra represent oil samples collected after 0, 3, and 8 h of sunlight exposure, respectively, at 26 ± 2 °C under moderate to high UV index (in the range 5–6) conditions. Compared to untreated control samples, the sunlight-exposed oil samples show the formation of numerous aldehyde signals, after 3 and 8 h of exposure. The signal assignments agree with those given in Table 4. Unassigned aldehyde signals are labeled with * and #.

Primary lipid oxidation products, such as hydroperoxides and hydroxides, are highly unstable and degrade further to form secondary oxidation products, including aldehydes, ketones, alcohols, and epoxides. Among these, aldehydes produce characteristic proton signals in the ^1^H NMR spectral region of 10.5–9.0 ppm, which are well separated from the signals of major acyl and glyceryl groups. At the initial time point (0 h of sunlight exposure), the ^1^H NMR spectra of the rapeseed, sunflower, sesame, and peanut oils showed negligible aldehyde signals, whereas those of the olive oil exhibited several aldehyde signals (Figure 2, 0 h). It is noteworthy that the olive oil sample had expired 10 days prior to the photooxidation experiments. The primary purpose of using the expired olive oil was to demonstrate the formation of lower amounts of aldehydes already present due to autoxidation, which may be one of the reasons the oil is deemed unsafe for consumption after expiration. Additionally, the experiments aimed to study the behavior of these pre-existing aldehydes during photooxidation. The identified aldehydes included the following: A: (*E*)-2-alkenals, B: (*E*,*E*)-2,4-alkadienals, C: 4,5-epoxy-(*E*)-2-alkenals, D: combined 4-hydroxy/4-hydroperoxy-(*E*)-2-alkenals, E: (*Z*,*E*)-2,4-alkadienals, F: n-alkanals, G: 4-oxo-(*E*)-2-alkenals, H: low-molecular-mass short-chain n-alkanals, such as propanal and n-butanal, and I: (*Z*)-2-alkenals (Table 4). These assignments were based on previously reported findings [10]. The pre-existing aldehydes in olive oil, predominantly (*E*)-2-alkenals and n-alkanals, were further amplified during photooxidation. After 3 h of sunlight exposure, new aldehyde proton signals were observed in all the oils compared to their respective control spectra (Figure 3, 3 h). The intensity of these signals increased significantly after 8 h of sunlight exposure, accompanied by the formation of additional aldehyde compounds (Figure 3, 8 h). The quantity of aldehydes that were generated after 8 h of sunlight exposure was notably higher in oils that were rich in polyunsaturated fatty acids, such as sesame, sunflower, and rapeseed oils, compared to olive and peanut oils. The aldehyde proton signal at 9.49 ppm, corresponding to (*E*)-2-alkenals, was most intense in the sesame oil, followed by the sunflower oil. In contrast, it was relatively less intense in the olive, rapeseed, and peanut oils. The sunflower oil exhibited the highest quantities of (*E*,*E*)-2,4-alkadienals, followed by the rapeseed and sesame oils, whereas the olive and peanut oils contained very low amounts. All oils showed significant generation of n-alkanals, as indicated by the aldehyde proton signal in the region of 9.76–9.73 ppm. Additionally, 4-oxo-(*E*)-2-alkenals and low-molecular-mass short-chain n-alkanals were generated in all oils, as indicated by the aldehyde proton signals in the chemical shift range of 9.81–9.77 ppm. The wide range of chemical shifts that were observed for the aldehyde proton signals clearly indicated the generation of diverse aldehyde-associated compounds during the light exposure of the oils. Although sunlight treatment is highly relevant to real-world applications, its primary limitation lies in the dependence on sunny and clear weather conditions, which makes it challenging to conduct experiments during winter or on cloudy days. Therefore, future studies would benefit from performing photodegradation experiments using UV lamps under controlled conditions. This approach would provide a deeper understanding of the long-term impact of light on the molecular composition of edible oils.

### 3.4. Generation of Aldehyde Compounds During Deep-Frying of Oils

Rapeseed, sunflower, sesame, and peanut oils were subjected to deep-frying in a commercial mini fryer at 190 ± 5 °C for 60 min, with chicken nuggets being intermittently fried to simulate commercial food preparation.

The ^1^H NMR spectra of the unheated control oil samples displayed negligible or minimal intensities of aldehyde proton signals. However, even 10 min of deep-frying at this high temperature resulted in the formation of new aldehyde compounds, accompanied by an increase in the intensity of the pre-existing aldehyde signals. After 60 min of continuous deep-frying, the ^1^H NMR spectra of all four oils showed a significant increase in the intensity of prior aldehyde signals and the generation of numerous new aldehyde compounds.

**Figure 4:**
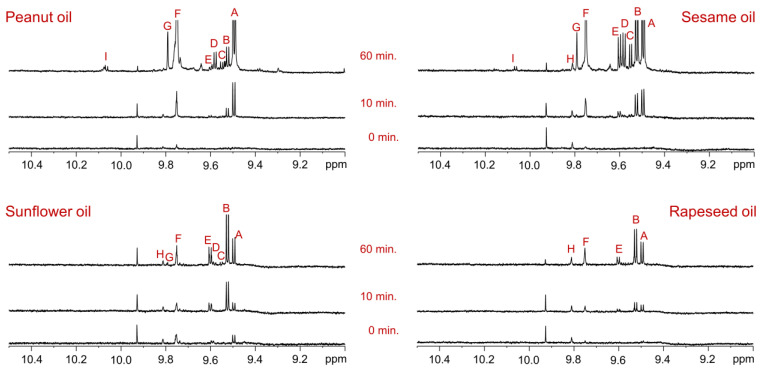
Aldehyde proton regions of ^1^H NMR spectra of oils, recorded in CDCl_3_ using an 800 MHz instrument. Each panel is labeled with the corresponding oil type. The bottom, middle, and top spectra represent oil samples collected at 0, 10, and 60 min of deep-frying, respectively, during intermittent frying of chicken nuggets at 190 ± 5 °C. The signal assignments agree with those given in Table 4.

Among the oils, the sesame oil exhibited the highest quantities of (*E*)-2-alkenals, (*E*,*E*)-2,4-alkadienals, 4,5-epoxy-(*E*)-2-alkenals, combined 4-hydroxy/4-hydroperoxy-(*E*)-2-alkenals, (*Z*,*E*)-2,4-alkadienals, n-alkanals, and 4-oxo-(*E*)-2-alkenals (Figure 4). Similarly, the sunflower and rapeseed oils demonstrated high abundances of (*E*)-2-alkenals, (*E*,*E*)-2,4-alkadienals, (*Z*,*E*)-2,4-alkadienals, and n-alkanals (Figure 4). Peanut oil generated significant levels of (*E*)-2-alkenals, (*E*,*E*)-2,4-alkadienals, combined 4-hydroxy/4-hydroperoxy-(*E*)-2-alkenals, n-alkanals, and 4-oxo-(*E*)-2-alkenals (Figure 4). These results can be attributed to the unique molecular composition of edible oils, which includes major and minor constituents such as saturated, monounsaturated, and polyunsaturated fatty acids, as well as minor components like tocopherols, phenolic compounds, sterols, carotenoids, and pigments such as chlorophyll [14,22]. The susceptibility of these components to deep-frying conditions likely contributed to the observed oxidation products.

These findings underscore that, regardless of the oil type or fatty acid composition, all oils are highly susceptible to thermal oxidation processes during prolonged exposure to high temperatures and repeated heating cycles. The presence of toxic aldehyde compounds in freshly used oils, even after a few minutes of deep-frying, suggests that their concentrations could increase several-fold if the oil is continuously heated for hours or days, posing significant health risks. This emphasizes the critical need to limit repeated oil usage to reduce the consumption of harmful oxidation products and ensure food safety.

## 4. Conclusions

This study highlights the significant oxidative transformations that occur in edible oils when exposed to sunlight and high-temperature deep-frying, providing critical insights into their degradation pathways and the formation of harmful compounds. Using high-field ^1^H NMR spectroscopy, we identified the generation of primary lipid oxidation products, such as hydroperoxides, and subsequent formation of diverse secondary oxidation products, including aldehydes and epoxides. These findings underscore the susceptibility of oils to thermal and photooxidation. The ^1^H NMR spectra revealed the rapid formation and accumulation of toxic aldehyde compounds under thermal and sunlight exposure, including 4-hydroperoxy-(*E*)-2-alkenals, 4-hydroxy-(*E*)-2-alkenals, and 4,5-epoxy-(*E*)-2-alkenals. To mitigate these risks, it is essential to adopt safer practices, such as limiting the reuse of oils in cooking, selecting oils with higher oxidative stability for prolonged heating, and avoiding direct light exposure during storage. Future studies should expand the sample set to include a broader range of oils and employ advanced multivariate statistical analyses to provide a more comprehensive understanding of the oxidative evolution of edible oils during long-term storage and common cooking processes.

## Figures and Tables

**Table 1:** The chemical shift assignments and multiplicities of the signals in the ^1^H NMR spectra of the olive, rapeseed, sunflower, sesame, and peanut oils, recorded in CDCl_3_. The signal numbers correspond to those assigned in Figure 1.

Signal	Chemical Shift (ppm) and Multiplicity	Structural Group Assignment	
1	0.90–0.85 (t, 7.1 Hz)	–C**H_3_**	Terminal methyl group of all FAs, with the exception of unsaturated ω-3 FAs
2	0.98–0.95 (t, 7.5 Hz)	–C**H_3_**	Terminal methyl group of unsaturated ω-3 FAs (linolenic acyl group)
3	1.40–1.14 (m)	–(C**H_2_**)_n_–	Bulk acyl chain methylene groups of FAs
4	1.65–1.56 (m)	–OCO–CH_2_–C**H_2_**–	Acyl groups
5	2.07–1.97 (m)	–C**H_2_**–CH=CH–	Acyl groups
6	2.33–2.27 (td, 7.5, 4.9 Hz)	–OCO–C**H_2_**–	Acyl groups
7	2.78–2.74 (t, 7.1 Hz)	=CH–C**H_2_**–CH=	Diunsaturated ω-6 acyl groups
8	2.82–2.78 (t, 7.1 Hz)	=CH–C**H_2_**–CH=	Triunsaturated ω-3 acyl groups
9	4.13 (dd, 11.9, 6.0 Hz) and 4.28 (dd, 11.9, 4.3 Hz)	–C**H_2_**OCOR	Glyceryl groups
10	5.28–5.22 (m)	>C**H**OCOR	Glyceryl groups
11	5.41–5.28 (m)	–C**H**=C**H**–	Acyl groups

t: triplet; dd: doublet of doublets; td: triplet of doublets; m: multiplet.

**Table 2:** The molar percentages of various acyl groups of edible oils obtained from ^1^H NMR data.

	Ln or ω3 (%)	L (%)	O or MU (%)	S (%)
Peanut oil	0.2 ± 0.0	6.9 ± 0.1	77.2 ± 1.7	15.7 ± 1.8
Sesame oil	0.6 ± 0.0	40.5 ± 0.9	41.2 ± 1.0	17.7 ± 1.9
Sunflower oil	0.5 ± 0.0	60.5 ± 0.9	26.0 ± 0.5	13.0 ± 1.4
Rapeseed oil	7.4 ± 0.1	19.5 ± 0.3	63.9 ± 1.0	9.2 ± 1.4
Olive oil	0.7 ± 0.0	10.9 ± 0.2	69.5 ± 1.4	18.9 ± 1.6

Acyl groups: Ln, linolenic; ω-3, omega-3; L, linoleic; O or MU, oleic or monosaturated; S, saturated.

**Table 3:** The chemical shift assignments and multiplicities of olefinic and hydroperoxide proton signals in the ^1^H NMR spectra of the olive, rapeseed, sunflower, sesame, and peanut oils, recorded in CDCl_3_. The signal letters correspond to those assigned in Figure 2.

Signal	Chemical Shift (ppm) and Multiplicity	Structural Group Assignment	
a	5.56 (ddd, 15.2, 8.1, 3.7 Hz)	–C**H**=CH–CH=CH–	(*Z*,*E*)-conjugated dienes associated with hydroperoxides (-OOH)
b	6.55 (ddd, 15.2, 11.0, 4.0 Hz)	–CH=C**H**–CH=CH–	
c	5.99 (td, 11.0, 11.0, 5.4 Hz)	–CH=CH–C**H**=CH–	
d,h	5.49–5.43 (m)	–CH=CH–CH=C**H**–	
e	5.76–5.70 (m)	–C**H**=CH–CH=CH–	(*E*,*E*)-conjugated dienes associated with hydroperoxides (-OOH)
f	6.24 (dd, 15.2, 10.4 Hz)	–CH=C**H**–CH=CH–	
g	6.03 (dd, 15.2, 10.4 Hz)	–CH=CH–C**H**=CH–	
-	8.50–7.80 (bs)	–OO**H**	Hydroperoxide protons

dd: doublet of doublets; td: triplet of doublets; m: multiplet; ddd: doublet of doublet of doublets; bs: broad signals.

**Table 4:** The chemical shift assignments and multiplicities of aldehyde signals in the ^1^H NMR spectra of rapeseed, sunflower, sesame, and peanut oils recorded in CDCl_3_. The signal letters correspond to those assigned in Figure 3 and Figure 4.

Signal	Chemical Shift (ppm) and Multiplicity	Structural Group Assignment	
A	9.49 (d, 7.9 Hz)	–C**H**O	(*E*)-2-alkenals
B	9.52 (d, 8.0 Hz)	–C**H**O	(*E,E*)-2,4-alkadienals
C	9.55 (d, 7.8 Hz)	–C**H**O	4,5-epoxy-(*E*)-2-alkenals
D	9.58 (d, 7.8 Hz)	–C**H**O	Combined 4-hydroxy/4-hydroperoxy-(*E*)-2-alkenals
E	9.60 (d, 8.0 Hz)	–C**H**O	(*Z,E*)-2,4-alkadienals
F	9.75 (t, 1.7 Hz)	–C**H**O	n-alkanals
G	9.79 (bs)	–C**H**O	4-oxo-(*E*)-2-alkenals
H	9.81 (bs)	–C**H**O	Low-molecular-mass short-chain n-alkanals
I	10.06 (d, 8.1 Hz)	–C**H**O	(*Z*)-2-alkenals

d: doublet; t: triplet; bs: broad signal.

## Data Availability

The original contributions presented in this study are included in the article/Supplementary Material. Further inquiries can be directed to the corresponding author.

## Supplementary Materials

The following supporting information can be downloaded at: https://www.mdpi.com/article/10.3390/foods14030513/s1, Figure S1: The kitchen tools and materials used in the present study; Table S1: The quantities of fatty acids in all five edible oils as provided by the manufacturer.
