# Supplementary material for: Analysis of the Generation of Harmful Aldehydes in Edible Oils During Sunlight Exposure and Deep-Frying Using High-Field Proton Nuclear Magnetic Resonance Spectroscopy
Source: Foods. 2025 Feb 5;14(3):513. doi: 10.3390/foods14030513 (PMC11816481; doi:10.3390/foods14030513)
Supplement: Supplementary file 1 [file foods-14-00513-s001.zip › foods-3367239-supplementary.pdf]

Supporting Information

Analysis of the Generation of Harmful Aldehydes in Edible Oils During Sunlight Exposure and Deep-Frying Using High-Field Proton Nuclear Magnetic Resonance Spectroscopy

Anna Meike Freis and Sahithya Phani Babu Vemulapalli*

Institute for Chemistry and Biology of the Marine Environment (ICBM), School of Mathematics and Science, Carl von Ossietzky Universität Oldenburg, Ammerländer Heerstraße 114–118, 26129 Olden-burg, Germany.

*Correspondence:

sahithya.phani.babu.vemulapalli@uni-oldenburg.de; Tel.: +49-441-3798-3398

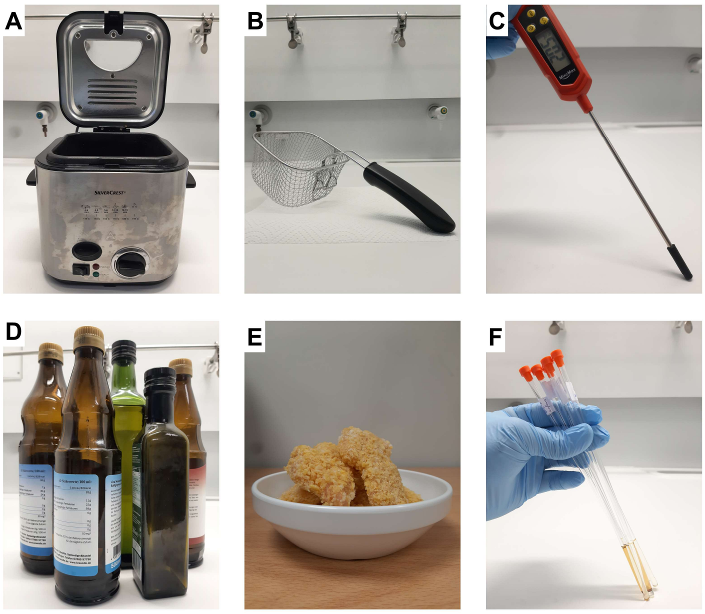

Figure S1: The kitchen tools and materials used in the present study. (A) A Silvercrest® Kitchen Tools mini deep fryer with temperature control. (B) Stainless steel deep fryer basket with handle. (C) A MixcMax kitchen thermometer (temperature range: -50 to +300 °C). (D) Olive, rapeseed, sunflower, sesame, and peanut oils in ambered/green-colored bottles. (E) Chicken nuggets used in deep frying. (F) 5 mm NMR tubes containing various oil samples dissolved in $CDCl_3$.

Table S1: The quantities of fatty acids in all five edible oils as provided by the manufacturer.

**Nutritional values/100 ml**	**FAs, g**	**PUFAs, g**	**MUFAs, g**	**Saturated FAs, g**
Peanut oil	92	-	-	14
Sesame oil	92	41	39	12
Sunflower oil	92	59	22	11
Rapeseed oil	92	24	61	7
Olive oil	100	-	79	13

FAs, fatty acids; PUFAs, polyunsaturated fatty acids; MUFAs, monounsaturated fatty acids.
